# Epidemiology and socioeconomic features of appendicitis in Taiwan: a 12-year population-based study

**DOI:** 10.1186/s13017-015-0036-3

**Published:** 2015-09-17

**Authors:** Kai-Biao Lin, K. Robert Lai, Nan-Ping Yang, Chien-Lung Chan, Yuan-Hung Liu, Ren-Hao Pan, Chien-Hsun Huang

**Affiliations:** School of Computer & Information Engineering, Xiamen University of Technology, Xiamen, 361024 China; Department of Computer Science and Engineering, Yuan Ze University, Taoyuan, 32003 Taiwan; Management Center, Keelung Hospital, Ministry of Health and Welfare, Keelung, 20147 Taiwan; Institute of Public Health, National Yang-Ming University, Taipei, 11221 Taiwan; Department of Information Management, Yuan Ze University, Taoyuan, 32003 Taiwan; Innovation Center for Big Data and Digital Convergence, Yuan Ze University, Taoyuan, 32003 Taiwan; Section of Cardiology, Cardiovascular Center, Far Eastern Memorial Hospital, New Taipei City, Taiwan; Department of Obstetrics & Gynecology, Taoyuan General Hospital, Ministry of Health and Welfare, Taoyuan, Taiwan

**Keywords:** Appendicitis, Appendectomy, Epidemiology, Socioeconomic status

## Abstract

**Introduction:**

This paper presents an epidemiologic study of appendicitis in Taiwan over a twelve-year period. An analysis of the incidence in the low-income population (LIP) is included to explore the effects of lower socioeconomic status on appendicitis.

**Methods:**

We analyzed the epidemiological features of appendicitis in Taiwan using data from the National Health Insurance Research Database (NHIRD) from 2000 to 2011. All cases diagnosed as appendicitis were enrolled.

**Results:**

The overall incidences of appendicitis, primary appendectomy, and perforated appendicitis were 107.76, 101.58, and 27.20 per 100,000 per year, respectively. The highest incidence of appendicitis was found in persons aged 15 to 29 years; males had higher rates of appendicitis than females at all ages except for 70 years and older. Appendicitis rates were 11.76 % higher in the summer than in the winter months. A multilevel analysis with hierarchical linear modeling (HLM) revealed that male patients, younger patients (aged ≤14 years), and elderly patients (aged ≥60 years) had a higher risk of perforated appendicitis; among adults, the incidence increased with age. Moreover, the risk of perforation was higher in patients with one or more comorbidities. LIP patients comprised 1.25 % of the total number of patients with appendicitis from 2000 to 2011. The overall incidence of appendicitis was 34.99 % higher in the LIP than in the normal population (NP), and the incidence of perforated appendicitis was 40.40 % higher in the LIP than in the NP. After multivariate adjustment, the adjusted hospital costs and length of hospital stay (LOS) for the LIP patients were higher than those for the NP patients.

**Conclusions:**

Appendicitis and appendectomy in Taiwan had similar overall incidences, seasonality patterns, and declining trends compared to numerous previous studies. Compared to NP patients, LIP patients had a higher risk of appendicitis, longer LOS and higher hospital costs as a result of appendectomy.

## Introduction

Appendectomy is one of the most common operations worldwide [1]. Although numerous epidemiological studies on appendicitis have been conducted, most have focused on Western populations [2–7]; relatively few epidemiological studies have focused on appendicitis in Asian populations. Lee et al. [8] reported the epidemiological features and lifetime risk of appendicitis and appendectomy in South Korea using epidemiological data from 2005 to 2007. However, considering the relatively short observation period, determining long-term trends was challenging. In addition, several studies have been conducted in Taiwan regarding the epidemiological features of appendicitis [9–17]. These studies were chiefly concerned with the monthly variation in the incidence of acute appendicitis [11], the volume-outcome relationship of acute appendicitis [13], trend differentials in the incidence of ruptured appendicitis between rural and urban populations [15], and a comparison of the perforation rate of acute appendicitis between nationals and immigrants [16]. No comprehensive study has evaluated the epidemiology of appendicitis in Taiwan from 2000 to 2011. Furthermore, only a few studies have paid attention to the effect of socioeconomic status (SES) on appendicitis, particularly studies focusing on the low-income population (LIP) [18].

We performed a comprehensive study to investigate the epidemiological features of age, gender, comorbidities, readmission, length of hospital stay (LOS), hospital cost, incidences, seasonal variation and the effect of lower SES on appendicitis and appendectomy. We also compared the differences in adjusted costs and LOS for appendicitis between the LIP and normal population (NP). A multilevel analysis with hierarchical linear modeling (HLM) was performed using data from all appendicitis patients to assess the odds ratio of the occurrence of perforated appendicitis. The data were retrieved from the National Health Insurance Research Database (NHIRD) for all years from 2000 to 2011.

## Methods

### Data source

Taiwan launched the single-payer National Health Insurance (NHI) program in 1995; by 2000, the NHI coverage rate had expanded to 96.16 % of the Taiwanese population, and by 2011, coverage had reached 99.88 %. All eligible enrollees can access health care services from most clinics and hospitals by making a small copayment [19]. The National Health Insurance Bureau (NHIB) established a nationwide research database, which included nationwide population-based data with high quality control and representation. The NHIRD includes various data subsets, such as inpatient expenditures by admissions (DD), details of inpatient orders (DO), ambulatory care expenditures by visits (CD), and details of ambulatory care orders (OO). In this study, the DD dataset was used for further analysis.

To evaluate temporal trends, the estimated population of Taiwan from 2000 to 2011 was used to calculate the annual incidences of appendicitis and appendectomy. For all other analyses, the mean annual incidence for the aforementioned years was determined by combining the annual discharges and using the Taiwan census data as the denominator, which are created and maintained by the Taiwan Department of Household Registration of the Ministry of the Interior.

### Data protection and permission

To protect patient privacy, all personal information was encrypted using a double scrambling protocol. Before using the NHIRD and its data subsets, all researchers signed a written agreement declaring that they had no intention of obtaining information that could violate the privacy of patients or care providers. This study was approved by the Institutional Review Board (IRB) of Taoyuan General Hospital, which has been certified by the Ministry of Health and Welfare in Taiwan (IRB Approval Number: TYGH103015). The study protocol was also evaluated by the NHI Research Institutes, which consented to the planned analysis of the NHIRD data (Agreement Numbers: NHIRD-103-160 and NHIRD-104-081).

### Study design

The NHIRD contains registration files and original claims data, including patient demographics, diagnoses, and treatment details related to inpatient and outpatient claims for reimbursement. Every claimant of the NHI program from 2000 to 2011 was included in the study population (22,276,672 persons in 2000, which increased to 23,224,912 persons by 2011). The registration and claims data of the study cohort were obtained from the NHIRD, and the various expenditure categories were established according to the DD. One exclusion criterion and two inclusion criteria were used to select cases that were admitted because of appendicitis or appendectomy. The exclusion criterion was patients who had undergone incidental appendectomy (ICD-9-CM procedure code of 47.1). The inclusion criteria were the following International Classification of Disease, Version 9 (ICD-9) code items: (1) diagnostic codes 540–543; and (2) procedure code 47.0.

The analysis included four steps: (1) identification of data sources and extracting data; (2) investigation of the epidemiological features of age, gender, comorbidities, readmission, LOS, hospital costs, incidences, seasonal variation and the effect of SES on appendicitis and appendectomy; (3) conversion of the extracted data to a comparable metric; and (4) application of statistical models to evaluate hazard ratios for the risk of perforation.

### Data definition

To investigate the incidence of appendicitis in Taiwan, International Classification of Diseases, Ninth Revision, Clinical Modification (ICD-9-CM) diagnostic codes were used. The major diagnostic codes for appendicitis were 540 (acute appendicitis), 541 (appendicitis unqualified), 542 (other appendicitis), and 543 (other diseases of the appendix). Furthermore, code 540 was further classified as 540.0 (acute appendicitis with generalized peritonitis), 540.1 (acute appendicitis with peritoneal abscess), and 540.9 (acute appendicitis without mention of peritonitis). The procedure code was defined as 47.0 (appendectomy, excludes incidental). Perforated appendicitis was considered for appendectomies revealing evidence of perforation, peritonitis, rupture, or abscess (ICD-9-CM diagnostic codes 540.0 and 540.1). The perforation ratio was defined as the ratio of the number of perforated appendicitis diagnoses to the number of appendectomies. The case-fatality ratio was defined as the percentage of patients with an appendectomy who died during hospitalization.

### Classification of LIP and NP

To evaluate the effect of socioeconomics, the enrolled subjects were divided into NP and LIP groups based on Taiwan’s Social Assistance Act criteria and registration in Taiwan’s NHI database [18]. Low-income households were defined as those with a monthly average per-member gross income of less than the monthly minimum living expense standard of that residence region. The minimum living expense standard was defined as 60 % of the average monthly disposable income for each region. The family property could not exceed the amount announced by the central or municipal authorities in the corresponding year [20]. This segment of the population was recorded as the fifth class insured in Taiwan’s NHI database [19]. The NP was all individuals who were not in the LIP.

### Statistical analysis

The descriptive statistics for comparing the baseline characteristics included the number of cases, percentages, annual incidences (per 100,000 individuals), and 95 % Confidence Interval (CI) for the estimated rates. The Analysis of Variance (ANOVA) was used to describe and compare the continuous variables among the various subgroups. Statistical significance was set at 0.05. To evaluate the risk factors for perforated appendicitis, a multiple logistic regression analysis was performed, and the Adjusted Odds Ratio (AOR) was calculated. Multilevel analysis (or the hierarchical linear modeling, HLM method) was used as an analytical strategy, which allowed the evaluation of group-level and individual-level factors [21]. The hypothesis and formulas of the HLM analysis used in the present study were as follows.

Level 1 HLM Model

1 $$ \begin{array}{l}Yij=\beta 0+\beta 1\times (gender)+\beta 2\times \left( age\  group\ 1\right)+\beta 3\times \left( age\  group\ 2\right)+\beta 4\times \left( age\  group\ 3\right)+\\ {}\beta 5\times \left( age\  group\kern0.62em 4\right)+\beta 6\times \left( comorbidities\ 1\right)+\beta 7\times \left( comorbidities\ 2\right)+\beta 8\times \left( regional\  hospital\right)+\\ {}\beta 9\times \left( medical\kern0.5em  center\right)+\beta 10\times (suburban)+\beta 11\times (readmission)+\gamma .\end{array} $$

Level 2 HLM Model

2 $$ \beta 0 = \gamma 00 + \gamma 01 \times (SES) + \mu 0. $$

To estimate the incidence for the various populations in each age group, we constructed a life table in 5-year age intervals using combined incidence data from 2000 to 2011. To compare the incidence of appendicitis during various months and seasons, months with fewer than 31 days were adjusted to fit a standard 31-day month. To reduce the effect of extreme data on the mean LOS and hospital cost values, 1 % maximum and 1 % minimum values were excluded from the raw data. All statistical analyses were performed using the Statistical Package for the Social Sciences (SPSS) for Windows (Version 18.0).

## Results

From 2000 to 2011, 294,544 patients were diagnosed with appendicitis (24,545/year on average). Of these, 53.09 % were male, 45.54 % were female, and the remaining 1.37 % of the patients had missing gender information. The median ages of the patients with appendicitis and perforated appendicitis were 35 years (23, 51) and 44 years (27, 61), respectively. As shown in Table 1, 3.98 % of the patients with appendicitis exhibited one comorbidity, and 0.36 % exhibited two or more comorbidities; 19.54 % of the patients chose a laparoscopic appendectomy. The proportions of patients residing in urban, suburban and rural areas were 85.72 %, 13.07 % and 1.22 %, respectively. We observed a higher proportion of patients residing in urban areas compared to suburban and rural areas. The proportions of patients hospitalized in medical centers, regional hospitals and district hospitals were 46.93 %, 33.39 % and 19.68 %, respectively. This result indicated that a large proportion of patients were more likely to choose medical centers and regional hospitals for better medical care. All of these demographic characteristics were similar between male and female patients; however, the ratio of readmission for complications was higher in male patients than in female patients (3.89 % vs. 2.27 %), and the overall case-fatality ratio of appendectomies was higher for male patients than for female patients (0.14 % vs. 0.09 %, Table 1).

**Table 1:** Demographic characteristics of patients with appendicitis in Taiwan from 2000 to 2011

Variable	Total(*n =* 294,544)		Male(*n =* 156,371)		Female(*n =* 134,141)		*P*
	*n*	%	*n*	%	*n*	%	
Age Stratum							< 0.001
0–14 y/o	38,222	12.98 %	23,382	14.95 %	14,803	11.04 %	
15–29 y/o	91,965	31.22 %	48,056	30.73 %	41,589	31.00 %	
30–44 y/o	78,384	26.61 %	41,305	26.41 %	35,528	26.49 %	
45–59 y/o	48,590	16.50 %	25,001	15.99 %	23,478	17.50 %	
60 y/o or more	37,383	12.69 %	18,627	11.91 %	18,743	13.97 %	
Comorbidities ^a^							< 0.001
0	281,756	95.66 %	149,510	95.61 %	128,227	95.59 %	
1	11,732	3.98 %	6,235	3.99 %	5,485	4.09 %	
≥ 2	1056	0.36 %	626	0.40 %	429	0.32 %	
Readmission for complication ^b^							< 0.001
No	285,359	96.88 %	150,293	96.11 %	131,091	97.73 %	
Yes	9,185	3.12 %	6,078	3.89 %	3,050	2.27 %	
Hospital Mortality							< 0.001
No	294,197	99.88 %	156,147	99.86 %	134,019	99.91 %	
Yes	347	0.12 %	224	0.14 %	122	0.09 %	
Operation Type							< 0.001
OA	223,145	80.46 %	119,052	80.88 %	100,278	79.41 %	
LA	54,178	19.54 %	28,147	19.12 %	26,007	20.59 %	
Hospital Level ^c^							< 0.001
District Hospital	58,303	19.68 %	30,513	19.40 %	26,160	19.38 %	
Regional Hospital	139,070	46.93 %	74,210	47.17 %	63,211	46.83 %	
Medical Center	98,946	33.39 %	52,585	33.43 %	45,597	33.78 %	
Area level							< 0.001
Urban	252994	85.72 %	133,743	85.34 %	115,859	86.20 %	
Suburban	38568	13.07 %	21,004	13.40 %	17,014	12.66 %	
Rural	3594	1.22 %	1,965	1.25 %	1,536	1.14 %	

*OA* Open Appendectomy *LA* Laparoscopic Appendectomy

A total of 4,032 records of appendicitis patients were missing information regarding gender

The denominator for “Operation Type” was the total number of patients who underwent a primary appendectomy (277,323)

^a^ Comorbidities were identified by referring to the ICD-9-CM codes, as described in Appendix C in [17]

^b^ Readmission for complication was defined as readmission with the diagnosis of a commonly encountered postoperative complication within 1 month after an appendectomy (Appendix B in [17])

^c^ In Taiwan, there are four types of accreditation (medical center, regional hospital, district hospital, and unaccredited hospital). Unaccredited hospital refers to clinic, special pharmacy, and home care organizations; they cannot treat appendicitis patients. Therefore, in the present paper, we separate hospitals into three groups by accreditation status: medical center, regional hospital, and district hospital

### Appendicitis

**Fig. 1:**
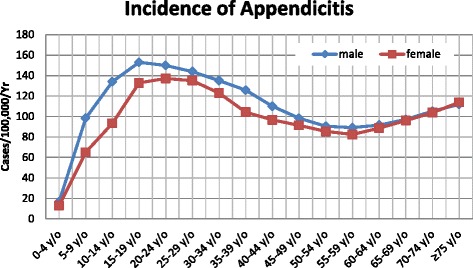
Annual incidence of appendicitis (per 100,000 people) in Taiwan according to age group and gender (2000–2011)

The overall incidence of appendicitis was 107.76 per 100,000 per year (95 % CI: 101.33–114.19), including 114.38 per 100,000 per year (95 % CI: 107.76–121) for males and 100.96 per 100,000 per year (95 % CI: 94.74–107.18) for females. The age-specific incidence of appendicitis displayed a similar pattern for both genders; the lowest incidence was observed in the 0-to-4-year age group, with an incidence of 16.62 per 100,000 per year (95 % CI: 14.10–19.15) for males and 12.90 per 100,000 per year (95 % CI: 10.67–15.12) for females. As shown in Fig. 1, the incidence gradually increased in subsequent age groups and peaked at 15-to-19-years (152.92 per 100,000/year) for males and at 20-to-24-years (137.20 per 100,000/year) for females. Subsequently, the incidence decreased gradually and reached the low point for the 55-to-59 year age group in both genders. The incidence then gradually increased again until it reached another peak at the age of 75 years and older. Overall, the 15-to-29-year age group was the highest risk group for both genders, and males exhibited a higher incidence at all ages except for 70 years and older (Fig. 1).

### Acute appendicitis

A total of 280,725 patients were diagnosed with acute appendicitis (23,394/year on average), which accounted for 95.31 % of the total number of patients diagnosed with appendicitis. The overall incidence of acute appendicitis was 102.69 per 100,000 per year (95 % CI: 96.41–108.97). The age-specific incidence of acute appendicitis exhibited a similar trend as that of appendicitis. The only difference was that the incidences of acute appendicitis in each age group were slightly lower than those of appendicitis, as acute appendicitis is a subcategory of appendicitis.

### Primary appendectomy

A primary appendectomy was defined as a non-incidental appendectomy. A total of 277,323 patients underwent an appendectomy from 2000 to 2011. Among these, 268,288 patients were diagnosed with appendicitis at discharge, and the remaining 9,035 patients were not diagnosed with appendicitis at discharge, indicating that they may have been misdiagnosed. The overall incidence of primary appendectomy was 101.58 per 100,000 per year (95 % CI: 95.34–107.82). The incidence of primary appendectomy was higher in males than in females (male-to-female ratio of 1.13:1), with values of 107.83 per 100,000 per year (95 % CI: 101.40–114.27) and 95.15 per 100,000 per year (95 % CI: 89.11–101.20) for males and females, respectively. The age-specific incidences of primary appendectomy were similar for both genders, but males exhibited higher rates at almost all ages; in the 60-to-69-year age group, females exhibited a slightly higher incidence than males.

### Perforated appendicitis

**Fig. 2:**
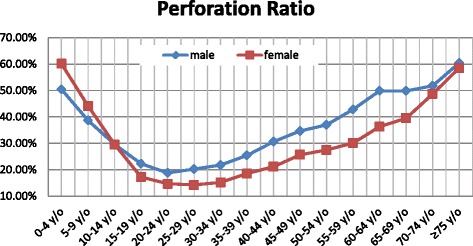
Perforation ratios (per 100,000 people) in Taiwan according to age group and gender (2000–2011)

A total of 74,326 patients exhibited appendiceal perforation, rupture, abscess, or generalized peritonitis. Among these, 58.28 % were males, 40.75 % were females, and the remaining 0.97 % of patients had missing gender information. The overall incidence of perforated appendicitis was 27.20 per 100,000 per year (95 % CI: 23.97–30.44). Male patients had a higher risk of having perforated appendicitis than female patients at all ages. The incidence by gender was 31.59 per 100,000 per year (95 % CI: 28.11–35.08) for males and 22.69 per 100,000 per year (95 % CI: 19.74–25.65) for females, respectively, with an overall male-to-female ratio of 1.39:1. The overall perforation ratio was 25.23 % (27.70 % for male patients and 22.58 % for female patients). The age-specific perforation ratios were similar for both genders; these ratios were higher in the older and younger patients but lower at intermediate ages, thus exhibiting a V-shaped pattern (Fig. 2).

A multilevel analysis with HLM was used to evaluate the individual effects (i.e., gender, age, comorbidities, hospital level, area level, and readmission) and the group effect (i.e., SES) on the incidence of perforated appendicitis. Male patients had a higher risk of suffering from perforated appendicitis than female patients. Compared to the 15-to-29-year age group, the younger patients (aged ≤14 years) and adults (aged ≥30 years and older) exhibited a higher risk of perforated appendicitis; among adults, the incidence of ruptured appendicitis increased with age (Table 2). In addition, the risk of perforation was higher in patients with one or more comorbidities, and this risk increased further as the number of comorbidities increased. The risk of perforation was higher in patients admitted to regional hospitals and medical centers than in patients admitted to district hospitals. Furthermore, the risk of ruptured appendicitis increased significantly in patients who were readmitted to a hospital for complications (AOR = 4.930, *p <* 0.001). Compared to the NP, the LIP exhibited a higher risk of ruptured appendicitis (AOR = 1.098, *p =* 0.016). These patterns were consistent in both genders (Table 2).

**Table 2:** Multilevel analysis (with HLM) of the risk factors for perforation among male and female patients with appendicitis in Taiwan (2000–2011)

Variable	Total			Male			Female		
	β value	AOR	*P*	β value	AOR	*P*	β value	AOR	*P*
Gender									
Female^c^	1.0								
Male	0.264	1.302 (1.279,1.324)	< 0.001						
Age(years)^a^									
0–14 y/o	0.779	2.179 (2.119,2.241)	< 0.001	0.620	1.858 (1.793,1.927)	< 0.001	1.048	2.851 (2.727,2.980)	< 0.001
15–29 y/o^c^	1.0			1.0			1.0		
30–44 y/o	0.283	1.327 (1.295,1.361)	< 0.001	0.302	1.353 (1.310,1.397)	< 0.001	0.268	1.307 (1.257,1.359)	< 0.001
45–59 y/o	0.758	2.134 (2.078,2.191)	< 0.001	0.774	2.167 (2.092,2.245)	< 0.001	0.758	2.134 (2.049,2.222)	< 0.001
≥60 y/o	1.290	3.633 (3.532,3.737)	< 0.001	1.234	3.433 (3.304,3.568)	< 0.001	1.336	3.805 (3.647,3.969)	< 0.001
Comorbidities^a^									
0^c^	1.0			1.0			1.0		
1	0.353	1.424 (1.367,1.483)	< 0.001	0.367	1.443 (1.365,1.525)	< 0.001	0.335	1.398 (1.316,1.484)	< 0.001
≥2	0.529	1.697 (1.495,1.926)	< 0.001	0.473	1.604 (1.361,1.892)	< 0.001	0.607	1.834 (1.508,2.231)	< 0.001
Hospital Level^a^									
District Hospital^c^	1.0			1.0			1.0		
Regional Hospital	0.223	1.250 (1.219,1.281)	< 0.001	0.233	1.262 (1.222,1.304)	< 0.001	0.229	1.258 (1.210,1.308)	< 0.001
Medical Center	0.394	1.483 (1.444,1.523)	< 0.001	0.381	1.464 (1.413,1.516)	< 0.001	0.428	1.534 (1.472,1.598)	< 0.001
Area level^a^									
Urban^c^	1.0			1.0			1.0		
Suburban	0.038	1.038 (1.011,1.067)	0.007	0.044	1.045 (1.009,1.083)	0.015	0.008	1.008 (0.966,1.051)	0.713
Rural	0.269	1.308 (1.210,1.415)	< 0.001	0.251	1.285 (1.160,1.423)	< 0.001	0.256	1.292 (1.140,1.463)	< 0.001
Readmission^a^									
No^c^	1.0			1.0			1.0		
Yes	1.595	4.930 (4.712,5.159)	< 0.001	1.528	4.608 (4.361,4.870)	< 0.001	1.706	5.506 (5.087,5.961)	< 0.001
Socioeconomic status^b^									
Normal population^c^	1.0			1.0			1.0		
Low-income population	0.093	1.098 (1.018,1.184)	0.016	0.139	1.149 (1.038,1.273)	0.008	0.048	1.049 (0.936,1.176)	0.410

^a^Individual level. ^b^Cluster level. AOR: adjusted odds ratio.^c^: Reference group

A multivariate analysis was conducted after adjusting for age, gender, comorbidities, hospital level, area level, readmission, and socioeconomic status

### Utilization of care: LOS and hospital cost

**Fig. 3:**
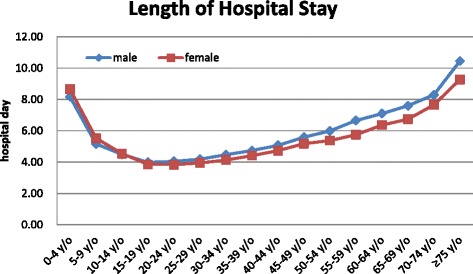
Length of hospital stay (per 100,000 people) for appendicitis in Taiwan by age group and gender (2000–2011)

The period between admission and discharge was defined as the LOS (measured in days). For patients who were discharged on the day of admission, the LOS was recorded as 1 day [17]. From 2000 to 2011, the estimated LOS for appendicitis was 1,510,007 days (125,833/year) in total. As shown in Table 3, the mean LOS values for appendicitis, acute appendicitis, and primary appendectomy were similar, whereas the LOS for perforated appendicitis was longer. The male-to-female ratio for the mean LOS values ranged from 1.01 to 1.05:1, which indicated that the mean LOS values were slightly higher for male patients than for female patients. The overall age-specific trend of LOS was a U-shaped pattern. The incidence was higher in the older and younger age groups but lower in the intermediate age group. The age-specific LOS for appendicitis displayed a similar pattern in both sexes, but males had higher rates at virtually all ages except the 0-14-year age group (Fig. 3). The mean LOS was 9.01 days for patients with one commodity and 12.25 days for patients with two or more commodities, which indicated that the mean LOS greatly increases for patients with one or more commodities compared to patients without commodity.

**Table 3:** The mean LOS and hospital cost for patients with appendicitis, acute appendicitis, primary appendectomy, and perforated appendicitis in Taiwan (2000–2011)

Variable	Gender	Appendicitis	Acute appendicitis	Primary appendectomy	Perforated appendicitis
Mean hospital stay ± SE (days)	Male	4.85 ± 0.01	4.77 ± 0.01	4.82 ± 0.01	7.63 ± 0.03
	Female	4.65 ± 0.01	4.56 ± 0.01	4.77 ± 0.01	7.44 ± 0.03
	Total	4.76 ± 0.01	4.67 ± 0.01	4.80 ± 0.01	7.55 ± 0.19
	Male–female ratio	1.04	1.05	1.01	1.03
Mean hospital cost ± SE (US$)	Male	1,052 ± 1	1,039 ± 1	1,091 ± 2	1,462 ± 5
	Female	1,030 ± 2	1,016 ± 1	1,120 ± 2	1,449 ± 6
	Total	1,042 ± 1	1,029 ± 1	1,104 ± 1	1,457 ± 4
	Male–female ratio	1.02	1.02	0.97	1.01

*SE* standard error of the mean

To reduce the effect of extreme data on the mean LOS and hospital cost values, the 1 % maximum and 1 % minimum values were excluded from the raw data

Hospital costs were calculated by adding all items in the hospital discharge summary together, including operation-associated costs and ward costs. Operation-associated costs included anesthesia and surgery fees and the costs of medical supplies used during the operation. Ward costs included surplus costs [17]. All costs were expressed in U.S. dollars (USD). In 2007, 1 USD was equivalent to approximately 32.64 New Taiwan dollars. As shown in Table 3, a positive association was observed between the hospital costs and the mean LOS: the longer the mean LOS, the higher the cost. The male-to-female ratio for the mean hospital cost ranged from 0.97 to 1.17:1; the cost was similar in both genders (Table 3).

### Seasonal variation

**Fig. 4:**
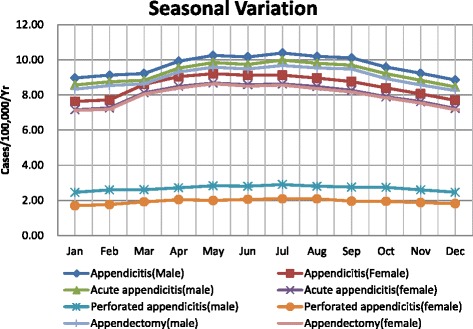
Monthly incidences of appendicitis, acute appendicitis, primary appendectomy, and perforated appendicitis in Taiwan (2000–2011)

The incidence of appendicitis revealed clear seasonality in both males and females, peaking in the summer and reaching troughs in the winter. Among males, the average incidence of appendicitis was 10.25 per 100,000 per month (adjusted to 31 days per month) in the summer and 9.04 per 100,000 per month in the winter. Therefore, the incidence of appendicitis in males in the summer was 11.76 % higher than the incidence in the winter. Among females, the average incidence was 9.10 per 100,000 per month in the summer and 7.78 per 100,000 per month in the winter. Therefore, the incidence of appendicitis in females in the summer season was 14.56 % higher than the incidence in the winter season. Similar seasonal variation was observed in the incidence of acute appendicitis and appendectomy. Slight seasonal variation was observed in the incidence of perforated appendicitis (the incidence was higher in the summer than in the winter), but the difference between the seasonal incidences was not as remarkable as those observed for appendicitis (Fig. 4).

### Socioeconomic status: LIP versus NP

The descriptive statistics of the NP and LIP with appendicitis are presented in Table 4. LIP patients accounted for 1.25 % of the total number of patients with appendicitis. The proportion of LIP patients gradually increased from 2000 to 2011.

**Table 4:** Descriptive statistics of the sample population for LIP and NP patients with appendicitis from Taiwan’s NHIRD (2000–2011)

Year	SUM	Normal population		Low-income population	
		*n*	%	*n*	%
2000	27,048	26,839	99.23 %	209	0.77 %
2001	27,941	27,677	99.06 %	264	0.94 %
2002	27,480	27,188	98.94 %	292	1.06 %
2003	25,099	24,819	98.88 %	280	1.12 %
2004	24,828	24,540	98.84 %	288	1.16 %
2005	23,686	23,401	98.80 %	285	1.20 %
2006	23,057	22,728	98.57 %	329	1.43 %
2007	23,122	22,831	98.74 %	291	1.26 %
2008	23,140	22,807	98.56 %	333	1.44 %
2009	23,365	23,013	98.49 %	352	1.51 %
2010	23,239	22,855	98.35 %	384	1.65 %
2011	22,586	22,212	98.34 %	374	1.66 %
Sum	294,591	290,910	98.75 %	3,681	1.25 %

The total number of patients (294,544) was smaller than the sum of the number of NP and LIP patients (294,591) because some patients belonged to different SES groups when they were readmitted to the hospital at different times

**Fig. 5:**
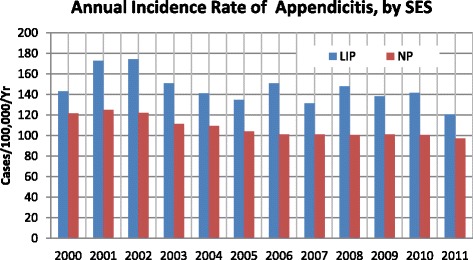
Annual incidence of appendicitis in Taiwan according to socioeconomic status (2000–2011)

**Fig. 6:**
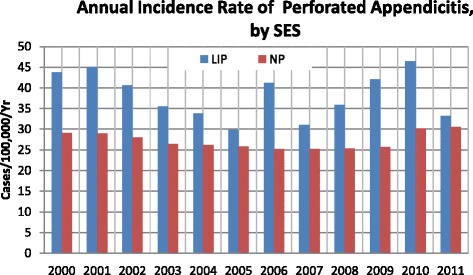
Annual incidence of perforated appendicitis in Taiwan according to socioeconomic group (2000–2011)

The overall incidence of appendicitis was 145.46 per 100,000 per year (95 % CI: 137.99–152.93) for the LIP, which was 34.99 % higher than the NP incidence of 107.76 per 100,000 per year (95 % CI: 101.33–114.19). The overall incidence of appendicitis for the LIP exhibited an annual wave-like trend but decreased in overall. Moreover, the annual incidence of appendicitis for the LIP was higher than the incidence for the NP (Fig. 5). The overall incidence of perforated appendicitis was 38.19 per 100,000 per year (95 % CI: 34.36–42.02) and 27.20 per 100,000 per year (95 % CI: 23.97–30.44) for the LIP and the NP, respectively, with an LIP-to-NP ratio of 1.40:1. Also displaying a wave-like trend, the annual incidence of perforated appendicitis was higher for the LIP than for the NP (Fig. 6).

The mean LOS for the LIP patients with appendicitis was 5.34 ± 0.09 days, which was 13.14 % higher than the LOS for the NP patients with appendicitis (4.72 ± 0.01 days). The mean hospital cost for the LIP patients with appendicitis was 1,157 ± 14 USD, which was 5.86 % higher than the hospital cost for the NP patients with appendicitis (1,093 ± 1 USD). Table 5 presents the differences in the adjusted costs and LOS between the LIP and NP patients stratified by various determinants. The coefficients in the multiple linear regression models represent the differences in specific outcomes between the target and reference groups. For example, the cost for the male LIP patients was higher by 37 ± 13 USD (*p* = 0.004) than the cost for the male NP patients. After multivariate adjustment, the adjusted costs for the female LIP patients were significantly higher than the costs for the female NP patients (96 ± 14 USD, *p* < 0.001) for the 30-to-44-year, 45-to-59-year, and 60 years and older age groups (144 ± 17, 274 ± 31, and 234 ± 48 USD, *p* < 0.001, respectively) and in the patients with perforated appendicitis (133 ± 2 USD, *p* < 0.001). The adjusted LOS for the male LIP patients was significantly longer than the LOS for the male NP patients (0.57 ± 0.009, *p* < 0.001) in the 45-to-59-year and 60 years and older age groups (1.39 ± 0.19 and 1.83 ± 0.26, *p* < 0.001, respectively) and in the patients with perforated appendicitis (0.68 ± 0.15, *p* < 0.001) (Table 5).

**Table 5:** Subgroup analysis with a multiple linear regression analysis to compare the differences in hospital costs (USD) and LOS (days) between LIP and NP patients

Stratified variables	Hospital cost (USD)			LOS (days)		
	SES (LIP versus NP)			SES (LIP versus NP)		
	Coefficient	SE	*P*	Coefficient	SE	*P*
Gender						
Male	37	13	0.004	0.57	0.09	< 0.001
Female	96	14	< 0.001	0.19	0.08	0.013
Age (years)						
0–14 y/o	−40	18	0.031	−0.20	0.12	0.086
15–29 y/o	45	11	< 0.001	0.02	0.07	0.819
30–44 y/o	144	17	< 0.001	0.34	0.06	< 0.001
45–59 y/o	274	31	< 0.001	1.39	0.19	< 0.001
60 y/o or more	234	48	< 0.001	1.83	0.26	< 0.001
Perforated appendicitis						
No	37	8	< 0.001	0.26	0.05	< 0.001
Yes	133	25	< 0.001	0.68	0.15	< 0.001

A multivariate analysis was conducted after adjusting for age, gender, hospital level, and comorbidities

*LIP* low-income population *NP* normal population *SE* standard error

### Temporal trends from 2000 to 2011

**Fig. 7:**
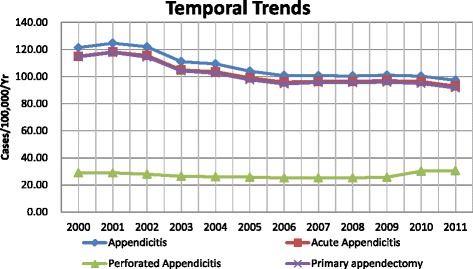
Temporal trends of appendicitis, acute appendicitis, perforated appendicitis, and appendectomy in Taiwan (2000–2011)

From 2000 to 2011, with the exception of a slightly higher incidence in 2001 compared to 2000, a clear downward trend was observed for the overall annual incidence of appendicitis. In 2000, the incidence was 121.41 per 100,000 per year (95 % CI: 114.58–128.23), and in 2011, the incidence was 97.24 per 100,000 per year (95 % CI: 91.14–103.35), reflecting a decrease of 19.9 % during the 12-year study period. Similar temporal trends were also observed regarding the incidence of acute appendicitis and primary appendectomy, with decreases from 2000 to 2011 of 19.2 % and 20.1 %, respectively. The overall incidence trend for perforated appendicitis displayed a gradual decrease from 2000 to 2009 and increased from 2009 to 2011 (Fig. 7).

## Discussion

Previous studies have provided various definitions of appendicitis. For example, several studies have lumped patients undergoing appendectomy with patients diagnosed with appendicitis [22, 23, 1]. David et al. [3] proposed that a patient with a positive primary appendectomy was considered to have acute appendicitis; thus, these terms were used interchangeably in their studies. Lee et al. [8] defined appendicitis as acute appendicitis (K35), other appendicitis (K36), and unspecified appendicitis (K37) according to the ICD-10. The definition used in the present study was similar to that defined by Lee et al., where a diagnosis of appendicitis was used regardless of whether the patient underwent an appendectomy. This definition can easily distinguish between appendicitis, acute appendicitis, and appendectomy but may slightly increase the incidence of appendicitis compared to other definitions.

In this study, the overall incidence of appendicitis was 107.76 per 100,000 per year (95 % CI: 101.33–114.19), which is consistent with the previously reported values of 75 to 120 per 100,000 per year in Western populations [1, 23, 3, 22, 24, 5, 6] but lower than a value in a South Korean population (227.1 per 100,000 per year) [8]. An epidemiological feature of appendicitis is the marked incidence variation according to age and gender. For both genders, the highest rates were observed in participants aged 15 to 29 years; this finding differed slightly from several previous studies that reported the highest incidence in participants aged 10 to 19 years [3, 22, 8]. In addition, the incidence of appendicitis was higher in male patients, with an overall male-to-female ratio of 1.14:1. This ratio is lower than the ratios reported in a previous study [3], which ranged from 2.2:1 to 3.3:1. In several previous studies [3, 25], the incidence of appendicitis declined with age in adults; however, in our study, although the incidence of appendicitis declined from the age of 15 to 55 years, the incidence increased after 55 years for both genders, suggesting that the risk of appendicitis increases with age after 55 years. Other studies have also suggested an increased incidence in the 60 year and above population. For example, Roger et al. [23] reported an increased incidence of perforating acute appendicitis in the 60 year and above population for people of Asian or African descent (as shown in Figs. 3–4 in [23]). Lee et al. [8] reported an increased incidence in the 55–79-year-old male population (Fig. 1 in [8]). In general, the age patterns for the incidence of appendicitis varied by country; the reason remains unexplained and requires further in-depth study via clinical trials.

The overall incidence of appendicitis was decreased by approximately 20 % from 2000 to 2011. A declining trend of appendicitis has been reported in several previous studies [3, 26, 22, 5, 27], but the reasons for the trend remain unclear. David et al. [3] summarized several possible explanations that have been proposed by previous studies, including nutritional and dietary changes [28], the increased use of antibiotics [29], improvements in SES [30], and changes in patterns of infectious disease and immunity [26]. All of these explanations may be relevant for the declining incidence of appendicitis in Taiwan, but no causal associations have been demonstrated. Contrary to the declining trends, other studies have also reported an increasing trend for the incidence of appendicitis [6, 31, 8] and a constant incidence of appendicitis [8, 32].

During the observation period, the proportion of patients undergoing laparoscopic appendectomy (LA) (19.54 %) was lower than that of patients receiving open appendectomy (OA) for appendicitis. There may be three reasons for why only a small amount of patients underwent LA. First, some surgeons had a doubling in the utilization of laparoscopy for appendectomy between 1999 and 2003 [33], and some surgeons were not very skilled at LA surgery when it was first introduced to Taiwan. Second, many patients were unwilling to try new surgical options; although LA is a standard operation, OA is more conventional than LA. Finally, changes in the NHI payment policy may have had a significant impact on the selection of LA for patients in Taiwan. The claims for appendicitis were processed by case payments before December 31, 2009. Thus, some of the material costs of LA were not covered by the NHI payment. This portion of hospital expenses may have required payment by the patients themselves, which led to some patients not selecting LA for economic reasons. Fortunately, the payment claims for appendicitis were changed to Taiwan Diagnosis Related Groups (Tw-DRGs) since January 1, 2010, and all LA costs have been included in the NHI payment system. Although the overall proportion of patients undergoing LA was lower than that of patients undergoing OA in our research phase, the frequency of LA increased over time, from 0 % in 2000 to 54.77 % in 2011 (data not shown).

A multilevel analysis using HLM was performed using data from 294,544 patients to assess the odds ratio of the occurrence of perforated appendicitis. As shown in Table 3, male, younger, and elderly patients were at increased risk of being diagnosed with perforated appendicitis. In addition, the analysis revealed that the risk of perforated appendicitis was significantly greater for the LIP patients and for patients who were readmitted for complications. As shown in Fig. 2, the perforation ratio was correlated with age, with the highest ratio in elderly and young patients. A similar phenomenon, which is referred to as the “J-shaped” trend, has also been reported in several previous studies [3, 8, 34, 35]; our study revealed a V-shaped trend. David et al. [3] stated that this pattern reflects both the increased diagnostic difficulty and the less timely surgical intervention for persons in these extreme age groups.

Clear seasonal variation was observed in the incidences of appendicitis, acute appendicitis, and appendectomy for both genders; the incidences increased in the summer season and decreased in the winter season. This pattern has been observed in several previous studies [3, 22, 24, 8, 11]. Wei et al. [11] analyzed the relationship between the incidence of appendicitis and climatic factors, including ambient temperature, relative humidity, atmospheric pressure, rainfall, and hours of sunshine; they reported a positive correlation between ambient temperature and the incidence of appendicitis. Kaplan et al. [36] reported a significant effect of air pollution on the incidence of appendicitis in the summer season. Although several factors may contribute to the seasonal variation in the incidences of appendicitis and appendectomy, no single causative factor has been identified [23, 3, 8]. In addition, the present study observed a slight but consistent increase in the incidence of perforated appendicitis in the summer season, which is inconsistent with several previous studies [37, 8].

The incidences of appendicitis (34.99 %) and perforated appendicitis (40.40 %) for the LIP patients were significantly higher than those for the NP patients. The reason for this pattern remains unexplained and requires further in-depth study, and clinical trials should be conducted to determine the reasons for such differences in risk. Moreover, the present study revealed that the mean LOS for the LIP patients with appendicitis was higher than the LOS for the NP patients. This finding may be attributable to three factors. First, the LIP patients may have resided in more remote areas than the NP patients, thereby requiring additional travel to obtain medical care [15]. Treatment delays related to travel may increase disease severity when patients finally arrive at a hospital, thereby necessitating a longer LOS. This may also explain the higher incidence of perforated appendicitis that was observed in the LIP patients compared to the NP patients. Second, poor financial conditions may reduce quality of life; therefore, the constitution of LIP patients may be weaker than that of NP patients, which may require a longer recovery time after an appendectomy. Finally, because health care provisions in Taiwan do not require LIP patients to pay for hospital costs, LIP patients may not consider the limitations of higher costs for a longer LOS.

The NHIB has established a uniform system to control the quality of medical services and coding. When the medical services provided to beneficiaries by contracted medical care institutions are deemed to be incompatible with the provisions of the NHI Act by the Professional Peer Review Committee, the expenses thereof are borne by the contracted medical care institutions themselves. Otherwise, the Disputes Settlement Board, which was established under the NHI scheme, settles disputes that arise in cases that were approved by the insurer and in cases that were claimed by the insured, group insurance applicants, or contracted medical care institutions [38, 39, 13, 40]. Based on the above, the data acquisition quality of the present study can be considered reliable. However, the present data are still subject to limitations.

One limitation is common to other administrative and claimed database-based studies: we could not review individual patient medical records that contained clinical data, and all of the information was in the form of numbers or codes. Without reviewing the individual medical records of each patient to ensure that the records were coded precisely, there could be deviations between the codes and the actual severity of the disease. Nonetheless, because the same database has been applied in many other fields of study with numerous high-impact publications, we believe that this population-based national claims database can be recognized as reliable [41]. The other limitation is that the information regarding gender was missing for 4,030 records of patients with appendicitis between the years 2000 and 2004 (958 records in 2000, 955 records in 2001, 879 records in 2002, 816 records in 2003, and 422 records in 2004). Information regarding gender was absent for one record in both 2006 and 2010 but was complete for all other years. The missing information regarding gender did not affect the calculation of the overall incidence, which was unrelated to the gender of the participants, but certain deviations are possible when comparing the incidence in male and female patients at various ages. To resolve this problem, we calculated the number of records for male and female patients in each age group, and the number of male patients was then divided by the number of female patients to obtain the male-to-female ratio. Subsequently, the records of the same age groups without information regarding gender were randomly assigned to the male or female groups according to the obtained gender ratio. This solution retained the total number of records and ensured that the male-to-female ratio was relatively accurate; nevertheless, some deviation still persisted, which is a limitation of our study.

## Conclusions

The present study shows that the incidence of appendicitis in Taiwan is consistent with several previous studies on Western populations but lower than the reported value of a South Korean population. The results also show that appendicitis is more common in males and that the appendicitis rate is higher in the summer months than in the winter months. The incidences of appendicitis, acute appendicitis, and primary appendectomy decreased annually, whereas the incidence of perforated appendicitis did not exhibit a clear trend. The above patterns are consistent with the results of several previous studies. However, the highest incidence of appendicitis was found in persons aged 15 to 29 years, which is different than the highest incidence in the 10-to-19-year group that was obtained in previous studies. A crucial finding was that the overall incidence of appendicitis for the LIP patients was 34.99 % higher than the overall incidence for the NP patients, and the incidence of perforated appendicitis was 40.40 % higher in the LIP than in the NP patients, indicating a significant negative effect of lower SES on the incidence and management of appendicitis and appendectomy.

## Abbreviations

LIP: Low-income population
NP: Normal population
SES: Socioeconomic status
NHI: National Health Insurance
NHIB: National Health Insurance Bureau
NHIRD: National Health Insurance Research Database
LOS: Length of hospital stay
HLM: Hierarchical linear modeling
DD: Inpatient expenditures by admissions
DO: Details of inpatient orders
CD: Ambulatory care expenditures by visits
OO: Details of ambulatory care orders
IRB: Institutional review board
CI: Confidence interval
ANOVA: Analysis of Variance
AOR: Adjusted odds ratio
SPSS: Statistical package for the social sciences
